# Evaluation of Microbial Communities Associated With the Liquid and Solid Phases of the Rumen of Cattle Offered a Diet of Perennial Ryegrass or White Clover

**DOI:** 10.3389/fmicb.2018.02389

**Published:** 2018-10-08

**Authors:** Jenna M. Bowen, Matthew S. McCabe, Susan J. Lister, Paul Cormican, Richard J. Dewhurst

**Affiliations:** ^1^Animal & Grassland Research and Innovation Centre, Teagasc, Dunsany, Ireland; ^2^Institute of Biological, Environmental & Rural Sciences, Aberystwyth University, Aberystwyth, United Kingdom; ^3^SRUC, Edinburgh, United Kingdom

**Keywords:** rumen, microbiota, cattle, liquid phase, solid phase

## Abstract

Rumen microbiota plays an important role in animal productivity, methane production and health. Several different locations have been used to obtain rumen samples (i.e., liquid-phase samples, solid-phase samples, buccal swabs) in previous studies. Here we assess differences in the rumen microbiota between solid- and liquid-phases of the rumen under differing dietary conditions (white clover vs. perennial ryegrass); there were 4 sample types: liquid-associated/grass (LG), solid-associated/grass (SG), liquid-associated/clover (LC), and solid-associated/clover (SC). Four Holstein-Friesian cows were strip grazed on pure stands of perennial ryegrass or white clover in a change-over design experiment with 3 periods (each lasting for 3 weeks). Solid- and liquid- phase microbes were obtained following total rumen evacuation on the penultimate day of each period. DNA was extracted and multiplexed libraries sequenced using 16S next generation sequencing (Illumina MiSeq). Demultiplexed sequences underwent quality control and taxonomic profiles were generated for each sample. Statistical analysis for the effects of diet and phase was conducted both overall [using non-metric multidimensional scaling (NMDS) and diversity indices] and for individual taxa. Separation of both diet and phase was observed NMDS, with significant effects of diet (*P* < 0.001) and phase (*P* < 0.001) being observed. Regardless of diet, *Prevotella* was most abundant in the liquid samples. When assessing differences between phases, the majority of statistically significant taxa (predominantly from Archaea and the order Clostridiales) were found at higher relative abundances in solid-phase samples. Diversity (Shannon Index) was lower in the liquid-phase samples, possibly because of the higher relative abundance of *Prevotella*. A presence vs. absence approach, followed by Chi-squared testing, was adopted. Differences between phases (LG vs. LC, LC vs. LG, SG vs. SC, and SC vs. SG) and differences between phases for the clover diet (LC vs. SC and SC vs. LC) were significant (*P* < 0.001); differences between phases for the grass diet were non-significant. Sampling technique has a profound impact on reported microbial communities, which must be taken into consideration, particularly as archaea may be underestimated in the liquid-phase.

## Introduction

The rumen microbiota plays an important role in animal physiology, with major effects on productivity. The diverse microbiota are responsible for degradation of complex carbohydrates, production of volatile fatty acids and synthesis of microbial protein. An understanding of microbial communities is essential for determining links to feed efficiency and methane production.

There are many factors that affect microbial communities in the rumen, including age (Jami et al., 2013), diet (Petri et al., 2013) and breed (Paz et al., 2016), as well as variation between individual animals (Henderson et al., 2015). Diet has a significant impact on rumen microbial communities and fermentation patterns, which in turn have significant effects on health, feed efficiency and methane production (Auffret et al., 2017), for example, and increased relative abundance of *Prevotella* spp. (carbohydrate utilizing bacteria) have been found in cattle offered a high-energy, low-forage diet (Carberry et al., 2012).

There is significant variation in sampling techniques e.g., liquid sample, solid bolus, buccal swabs, (Tapio et al., 2016; Ji et al., 2017) and this might affect interpretation of the role of the microbiome. Several studies showed differences in the rumen microbiota from liquid and adherent (solid) fractions of the rumen (Jewell et al., 2015: Veneman et al., 2015; de Mulder et al., 2017). Further, it has been suggested that the liquid-phase microbiome may be more diverse (Jewell et al., 2015). These previous studies have typically assessed the effect of ruminal phase on microbiota from housed cattle indoors, or fed a total mixed ration (forage and concentrate based diet), or both (Veneman et al., 2015; Tapio et al., 2016; de Mulder et al., 2017; Ji et al., 2017). To our knowledge, no other study has assessed the effect of rumen phase on microbial populations from grazing cattle at pasture on two contrasting diets (perennial ryegrass and white clover). These two contrasting diets were used as legumes (white clover used in this study) are a good source of protein relative to grasses, whilst grasses have higher levels of water soluble carbohydrates. Due to the lower fiber and higher protein content of white clover, grazing this legume allows for higher rates of digestion relative to grass. The design and sampling methods adopted here compare extremes of herbage (perennial ryegrass vs. white clover) under actual grazing conditions. Thus, the measurements here reflect differences in feeding and rumination behavior on rumen function and in turn the microbiota.

The aim of this study was to assess differences in microbial communities (i) between solid- and liquid- phases of the rumen and (ii) between differing dietary conditions – with cows grazing white clover or perennial ryegrass.

## Materials and Methods

### Animal Study and Sample Collection

Full details of the animal study can be found in McCartney et al. (2014). This study was conducted at Aberystwyth University in accordance with the UK Animal (Scientific Procedures) Act (1986). The study was run under a Home Office project license issued before local ethical review processes were implemented in April 1999. The operation of the study was monitored by the Local Ethical Review Group of IGER (Institute of Grassland & Environmental Research). In brief, 4 lactating Holstein-Friesian cows fitted with rumen cannulae were strip grazed on pure stands of either perennial ryegrass (Lolium perenne cv. Fennema) or white clover (Trifolium repens cv. AberHerald) in a changeover design with three 3-week periods. Note that the design of this study meant that each animal had a different dietary history. Cows were milked twice daily (8 am and 4 pm) and received 2 kg/head of a proprietary concentrate feed at each milking. Chemical composition of forages and concentrates are found in **Table 1**. Rumen contents were obtained by total rumen evacuation at 9 am on the penultimate day of each period.

**Table 1:** Chemical composition of feeds used in this study (McCartney et al., 2014).

Component (g/kg DM, unless stated otherwise)	Perennial ryegrass	White clover	Concentrates
DM (g/kg)	113	94.0	870
OM	899	886	914
NDF	486	231	279
ADF	269	211	142
Ether extract	30.6	19.9	54.1
Crude protein (N × 6.25)	216	309	200
Starch	–	–	283
Neutral cellulase gammanase digestibility	–	–	823
Digestible organic matter	663	749	–
Water soluble carbohydrates	96.1	51.0	89.2


### Isolating Liquid and Solid Phase Microbes

Rumen contents were hand squeezed through 4 layers of cheesecloth to obtain approximately 1 L of liquid. Liquid associated microbes were obtained from this. Solid associated microbes were obtained by gently washing 500 g rumen contents (previously retained in cheesecloth) with physiological saline and hand squeezed twice to remove any remaining liquid associated microbes. Samples were then processed in a Stomacher 400 Circulator (Seward UK Ltd., Worthing, United Kingdom) to detach microbes from solid rumen contents. Differential centrifugation was used to remove feed particles at low speed (10 min at 500 × g) followed by a higher speed step (25 min at 25,000 × g) for both liquid and solid samples. Microbial pellets were washed twice with saline, freeze-dried and stored frozen at -20^o^C.

### DNA Extraction, Library Preparation and Next Generation Sequencing

DNA was extracted from freeze dried pellets using a slightly adapted version of the repeated bead beating and column filtration method (Yu and Morrison, 2004). DNA quality was assessed on an agarose gel, and quantified using Nanodrop 1000 spectrophotometer (Thermo Fisher, United States). Library preparation was carried out using PCR amplification of the hypervariable (V4) region of the 16S rRNA gene. PCR amplification was carried out using barcoded 16S Illumina primers containing 12 bp barcodes (515F/806R rcbc; Caporaso et al., 2011, 2012), Q5 Hot Start-High Fidelity DNA Polymerase and High GC Content Enhancer (New England Biolabs Inc., United States). Cycle conditions were 94°C (2 min), followed by 30 cycles of 94°C (10 s), 68°C (20 s), and 72°C (1 min). Libraries were immediately purified using the QIAquick PCR Purification Kit (Qiagen, Germany) and quantified using a Nanodrop 1000 (Thermo Scientific, United States). Each sample was combined in equimolar concentrations into a single pool. The pool was gel purified using the QIAquick Gel Extraction Kit (Qiagen, Germany), and checked for size with a DNA1000 chip on an Agilent 2100 Bioanalyser (Aligent Technologies, United States). The pooled library was quantified by qPCR on an ABI7500 FAST real time qPCR machine (Life Technologies, United States) using the Universal qPCR master mix from the Kapa library quantification kit for Illumina platforms (Kapa Biosystems, United States). Pooled libraries were then diluted to 2 nM, denatured with sodium hydroxide, spiked with denatured PhiX version 3 library (Illumina, United States) (6:4 volume:volume, Pooled libraries:PhiX V3 library) and loaded into a 300 cycle version 2 MiSeq reagent cartridge which was run on an Illumina MiSeq (Illumina, United States).

### Sequencing Data Clean-Up

Raw sequence reads for all samples in the study were quality controlled using the BBduk^1^ Java package. This was used to trim low quality bases (<20 Phred score) from the 3′ end of sequence read pairs, remove adaptor contamination, and remove read pairs containing ambiguous bases. Read pairs with an insert size (length of template molecule) shorter than the sum of the lengths of read 1 and read 2 were merged into a single, longer read. Size selection of 253 bp ± 20 bp sequences was performed with an in-house Perl script^2^. Chimeric sequences were identified using usearch61 against the GreenGenes database^3^ and removed. OTUs were assembled using the open reference method (a combination of reference based and *de novo* methodologies) using usearch61 with a 97% similarity used to cluster reads into individual OTUs. Taxonomy was assigned to these OTUs using the RDP classifier (v2.2) and the GreenGenes database. Associated sequence files have been submitted to NCBI Sequence Read Archive (Accession no. PRJNA416715). Unassigned taxa (unassigned at any level) were removed. Abundance estimates were calculated by summing read counts of OTUs with identical taxonomic assignments from Kingdom to Genus taxonomic level (**Supplementary Table S1**). Samples were assigned to four groups: (i) liquid-associated microbes from cows fed white clover (LC), (ii) solid-associated microbes from cows fed white clover (SC), (iii) liquid-associated microbes from cows fed perennial ryegrass (LG), and (iv) solid-associated microbes from cows fed perennial ryegrass (SG). Samples were rarefied to the lowest read number (54,500) across all samples with Shannon diversities calculated at each iteration, the average of which was used as the Shannon index value (H).

### Statistical Analysis

NMDS ordination plots were created using the metaMDS() function in the VEGAN package (Community Ecology Package, V 2.5-2) of R Studio (V 3.4.3), in which OTUs were rarefied to the lowest sequence number. In order to assess the effects of diet, phase, period and interactions on microbial populations, permutational multivariate analysis of variance (PERMANOVA) was carried out using the adonis() function in VEGAN. Beta-diversity between all groups (i.e., LC, SC, LC, LG) was assessed using betadisper() function in VEGAN.

The effects of diet and phase on relative abundances of genera were estimated using the Kruskal-Wallis (non-parametric) test in the STAMP statistical package (V 2.1.3; Parks et al., 2015) with a Benjamini-Hochberg false discovery rate applied.

Shannon diversity indices were calculated in QIIME for each of the samples to assess both species evenness and richness. Data was visualized using box plots. Differences between phases (liquid vs. solid), diet (white clover vs. perennial ryegrass) and period (1, 2, or 3) were assessed using analysis of variance (GenStat V14, Payne et al., 2011).

Samples were assessed by a presence or absence method, in which two of the four sample types (LC, SC, LG, or SG) were compared, one having OTUs present in ≥4 samples and the other having 0 OTUs present, e.g., ≥4 samples in LC and 0 in SC. Chi-squared testing (GenStat V14; Payne et al., 2011) was used to compare frequency of OTUs in ≥ 4 or 0 samples between diets or fractions. The null hypothesis was no difference between treatments (or fractions) in numbers of OTUs in ≥4 or 0 samples, respectively.

## Results

**FIGURE 1:**
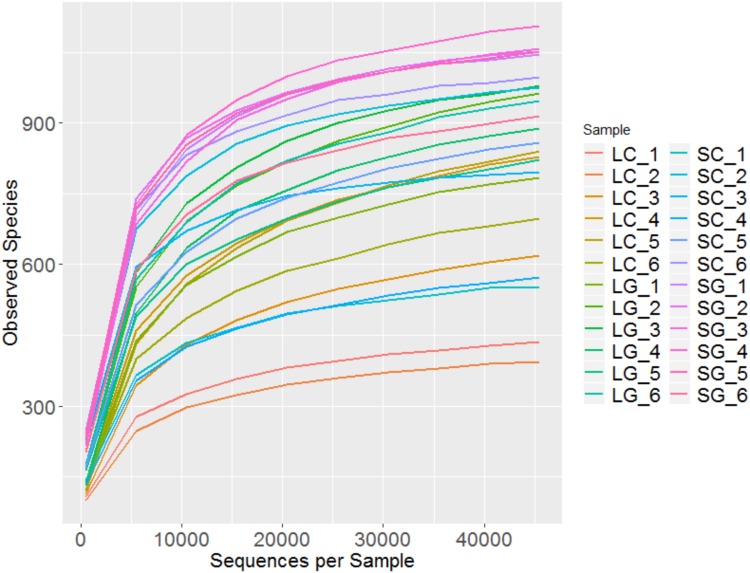
Rarefaction plot showing observed species from sample groups; LC (liquid clover), LG (liquid grass), SC (solid clover), SG (solid grass).

Overall 2,077,290 reads were generated which reduced to an average (± standard deviation) of 83,224 ± 12,620 reads per sample after filtering. Rarefaction plots (**Figure 1**) confirm that sequencing was performed to a sufficient depth.

### Community Structure

Bacteroidetes was the most abundant phylum in liquid samples [53.1% (s.d. 12.7) for LC and 61.5% (s.d. 15.4) for LG], followed by Firmicutes, which accounted for 38.1% (s.d. 15.8) and 27.9% (s.d. 11.4) of OTUs in the LC and LG, respectively. The reverse was seen in solid-phase samples: Firmicutes was the most abundant taxonomic group [59.8% (s.d. 6.0) and 49.8% (s.d. 5.7) for SC and SG, respectively], followed by Bacteroidetes [accounting for 25.9% (s.d. 6.5) in SC and 27.9% (s.d. 3.2) in SG]. Other phyla present, although at lower relative abundances (liquid; solid), included: Actinobacteria (4.0%; 3.4%), Fibrobacteres (1.8%; 5.2%), Tenericutes (1.2%; 1.6%), Spirochaetes (1.1%; 4.4) and Euryarchaeota (0.2%; 2.2%). Other phyla were present at <1%.

**FIGURE 2:**
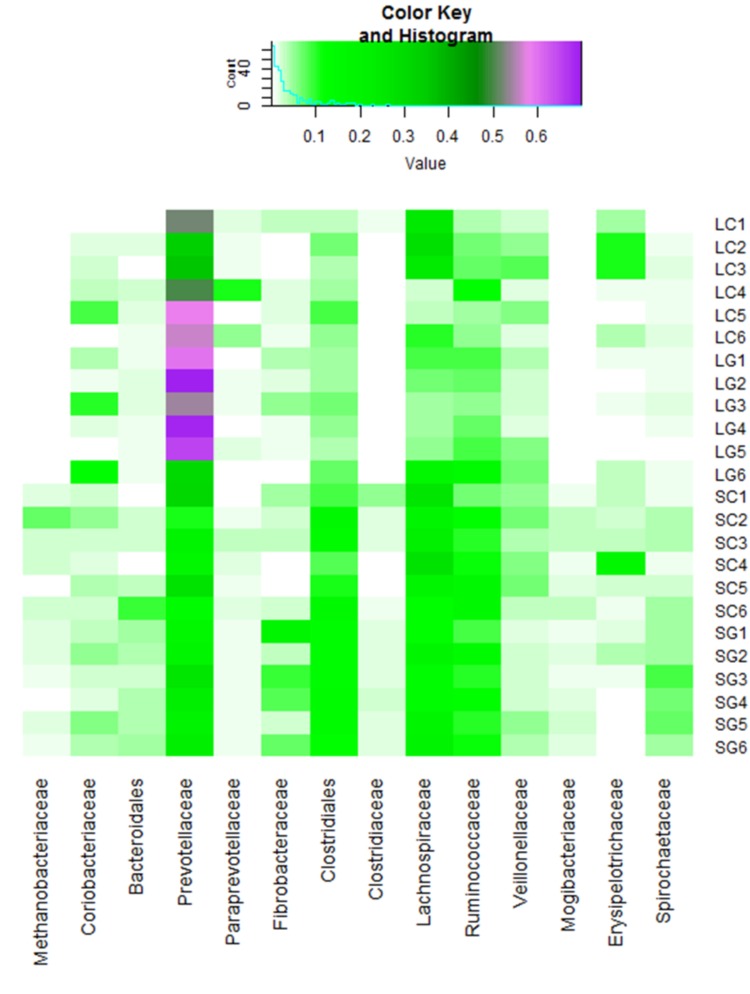
Heatmap of most abundant genera (relative abundance >1%).

*Prevotella* were the most abundant taxonomic group (genus level) within liquid-phase samples, 58.6% (s.d. 15.1) and 48.0% (s.d. 10.6) for LG and LC, respectively. *Prevotella* was also the most abundant genus within SG and SC samples, although at a lower level; 21.3% (s.d. 3.5) and 19.8% (s.d. 8.1), respectively (**Figure 2** and **Supplementary Figure S1**). Kruskal-Wallis test showed that the abundance of ten genera were significantly different when comparing microbes associated with LC and SC, including *Methanosphera*, *VadinCA11* and several from the Class Clostridia, all of which were higher in solid-phase samples. Seventeen genera were significantly higher in solid samples when comparing LG to SG, these included *Methanobrevibacter*, *Methanosphera* and several from the Class Clostridia. Two genera were significantly higher in clover when comparing SC and SG, these were *Methanosphera* and *Eubacterium*. No significant differences in abundances of genera were found when comparing LC and LG. When comparing liquid and solid samples, 33 genera were significantly different. All of these, except *Prevotella* and Order YS2, were higher relative abundance in solid-phase samples. Fifteen genera were significantly different when comparing grass to clover, and 47 genera were significantly different when assessing all samples at the same time. These results can be found in **Supplementary Table S2**.

**FIGURE 3:**
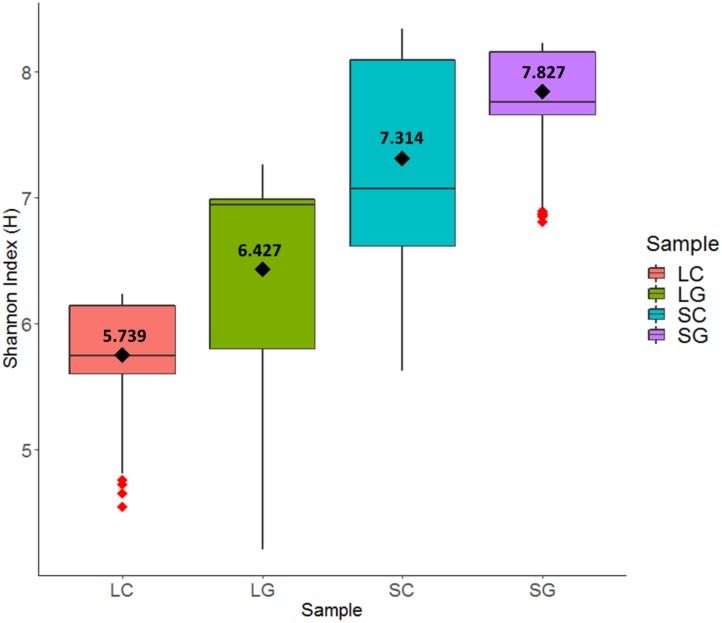
Differences in diversity using the Shannon Index (H): LC (liquid clover), LG (liquid grass), SC (solid clover), SG (solid grass).

Diversity within treatment (LC, SC, LG, SG), period (1, 2, 3), diet (white clover, perennial ryegrass) and phase (Liquid, Solid) were calculated using the Shannon Index (H). H values leveled off above 4,500 sequences per sample, so all H values were standardized to 4,500 sequences per sample with a corresponding Shannon index error value. Highest H values (s.d.) were observed for solid-phase samples 7.571 (0.604) compared to liquid-phase samples 6.083 (0.687) and this differences was statistically significant (*P* < 0.001). The same trend was also observed when comparing samples individually; SC [7.314 (0.236)], SG [(7.827 (0.236)], LC [5.739 (0.340)], and LG [6.427 (0.769)]. Perennial ryegrass samples had higher H values compared to white clover, 7.127 (0.902) versus 6.526 (0.974) respectively, but this was non-significant (*P* = 0.323). This is summarized in **Figure 3**.

### Overall Differences in Communities

**FIGURE 4:**
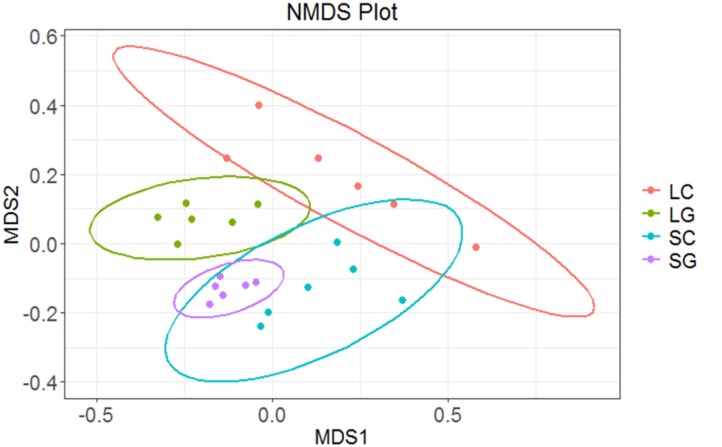
Differences in community structure between samples using NMDS plot: LC (liquid clover), LG (liquid grass), SC (solid clover), SG (solid grass).

Overall differences in community structure were assessed using NMDS. Separation of samples was observed for microbes associated with LG and SG (**Figure 4**). Greater variation was observed between LC samples, whilst SG samples showed the least variation, sample significantly effected beta-diveristy (*P* < 0.001). There were significant differences in microbial communities associated with diets (*R*^2^ = 0.187, *P* < 0.001) and phase (*R*^2^ = 0.155, *P* < 0.001), however period had no effect (*R*^2^ = 0.065, *P* = 0.118). An interaction between period and diet was observed (*R*^2^ = 0.063, *P* = 0.041), though there were no interactions between diet and phase, or period and phase (*P* > 0.05).

### Unique OTUs

A summary of the ‘presence vs. absence’ approach is shown in **Table 2**. The largest differences were seen for OTUs present in ≥4 LG diet samples and absent from all LC diet samples. Comparing the reverse of this situation (OTUs present in ≥4 LC samples and absent from all LG samples), only 17 OTUs were identified. The differences between these two groups in the number of unique OTUs were highly significant (**Table 2**; *P* < 0.001). Forty three unique OTUs were observed when comparing OTUs present in ≥4 SG and 0 SC samples, the reverse (≥4 SC; 0 SG) had 12 unique OTUs, with differences between these groups also being highly significant (**Table 2**; *P* < 0.001). ‘Presence vs. absence’ analysis was repeated for each diet (SG vs. LG; SC vs. LC). Only 2 OTUs were found to be unique between LG ( ≥ 4 OTUs) and SG (0 OTUs) samples; when considering the reverse situation (≥4 OTUs in SG; 0 OTUs LG samples), 6 were found to be unique; there were no significant difference when comparing these groups. No unique OTUs were identified when comparing ≥4 OTUs in LC and 0 OTUs in SC samples, the reverse analysis showed 21 unique OTUs between SC and LC samples, with differences between these groups being highly significant (*P* < 0.001; **Table 2**). The order Clostridiales, in particular families Lachnospiraceae and Ruminococcaceae, and family Prevotellaceae contained the majority of the unique OTUs, 61 and 39 OTUs, respectively out of a total of 167 OTUs. Clostridiales predominated amongst OTUs identified in ≥4 LG and 0 LC samples, the reverse of this (≥4 LC and 0 LG samples) and several in the ≥4 SC and 0 LC comparison. When comparing significant differences in OTUs present in ≥4 SG and 0 LG samples, the family Prevotellaceae dominated. A summary of significantly different taxa can be found in **Supplementary Table S3**.

**Table 2:** Summary of presence vs. absence analysis.

Present ≥4	Present 0	Number of OTUs	Chi-Squared	Significance (Chi-Squared)
LG	LC	67	28.93	*P* < 0.001
LC	LG	17		
SG	SC	43	17.47	*P* < 0.001
SC	SG	12		
LG	SG	2	1.07	NS
SG	LG	6		
LC	SC	0	14.00	*P* < 0.001
SC	LC	21		


*Samples were assessed by having OTUs present in ≥4 samples and the other having 0. Table also shows Chi-Squared results, all 1 degree of freedom. NS, non-significant.*

## Discussion

Previous research has focused on differences in the rumen microbiota between solid and liquid phases of the rumen, or dietary differences on housed cattle, typically offered a total mixed ration. This novel study assessed differences in phases of the rumen of dairy cows offered perennial ryegrass or white clover. Under these true grazing conditions differences in grazing behavior, ingestion, mastication and rumination between grasses and legumes will be reflected in rumen function and the rumen microbiota.

### Differences Between Diets

Differences in dietary treatment is often the main source of variability in rumen microbial communities (Petri et al., 2013), with different communities preferring particular substrates and metabolites. For example, *Prevotella* has previously been identified as a carbohydrate and nitrogen utilizing bacteria (Gasparic et al., 1995; Kim et al., 2017). The lack of significant diet effects on the relative abundances of *Prevotella* in the present study is likely caused by higher levels of protein in white clover counteracting the effects of higher water soluble carbohydrate in perennial ryegrass (**Table 1**).

Increases in relative abundances of Proteobacteria has been linked to imbalances in the gut microbiome (Shin et al., 2015). Higher levels of Proteobacteria have been found in cattle offered diets with a high level of concentrates (Auffret et al., 2017) – associated with indicators of rumen stress, such as low pH. In this study six genus level taxa from the phylum Proteobacteria were significantly increased in samples from animals offered the white clover diet relative to the perennial ryegrass. This suggests that the rumens of cattle fed white clover were under more stress, potentially associated with low rumen pH and/or the risk of bloat when feeding high levels of white clover. Pitta et al. (2016) also reported that Proteobacteria were increased in steers with wheat-infused frothy bloat.

Microbes present at low levels (below the detectable limit at this sequencing depth) are likely to be present within the rumen, but are only able to proliferate and increase in numbers when a more suitable substrate is introduced (e.g., perennial ryegrass compared to white clover diet). It must be noted that taxa reported in the current presence vs. absence analysis were only present at low relative abundances, ranging from 1 to 11,284, with an average of 59 reads per sample.

### Differences Between Phases

The majority of unique OTUs seen within diets and between phases (e.g., SC vs. LC) were members of the order Clostridiales, in particular the family Lachnospiraceae and Ruminococcaceae, with higher abundances in liquid-phase samples. In contrast, de Menezes et al. (2011) reported Lachnospiraceae are prevalent in solid samples. de Mulder et al. (2017) also reported that cellulolytic bacteria and secondary colonizers e.g., Lachnospiraceae, Ruminococcaceae and Christensenellaceae, were more abundant in solid-phase samples.

A second family, Coriobacteriaceae, was also found to be unique to solid-phase samples. Ruminococcaceae and Lachnospiraceae have been previously reported for their fibrolytic activity (Krause et al., 2003; Kong et al., 2010; Biddle et al., 2013). *Prevotella* was the most abundant genus in both liquid and solid samples, but was present at higher relative abundances in the liquid phase. This explains why Bacteriodetes predominated in the liquid-phase samples with Firmicutes predominating in the solid-phase samples. This is in agreement with other studies (Pitta et al., 2010; de Menezes et al., 2011; Patel et al., 2014; McCabe et al., 2015) where *Prevotella* was observed at higher relative abundances in the liquid fraction. These observations support suggestions of Prevotellaceae as polysaccharide metabolisers (Matsui et al., 2000; de Menezes et al., 2011). The liquid phase of the rumen includes dissolved sugars and other readily available substrates (de Mulder et al., 2017). In this study, methanogens were more abundant in solid phase samples. It must be noted that whilst de Mulder et al. (2017) found similar absolute abundances of methanogens in solid, adherent, crude rumen liquid, liquid and epimural samples, epimural samples had more methanogens relative to bacteria. The diversity of epimural samples varied greatly (some very low) relative to samples from other sites, and this is probably linked to the increase in relative abundance of methanogens. In the present study, the higher abundance of methanogens in solid phase samples was associated with higher community diversity. Methanogens are an important part of the solid adherent biofilm, attracted to feed particles by metabolites from cellulose-degrading bacteria. Methanogens are less abundant in the liquid phase, it being more difficult to form biofilms on free floating feed particles (Leng, 2014), particularly as methanogens are slow growing microbes. The action of rumen contraction means that the liquid phase of the rumen inoculates newly ingested solid material in the rumen mat. This explains why there is some degree of similarity between solid- and liquid-phase communities and, in particular, the near absence of OTUs that are present in liquid-phase samples and absent from solid-phase samples (**Table 2**).

Of the significantly different genera nearly all (with the exception of *Prevotella*) were more abundant in the solid-phase samples - leading to higher diversity. Jewell et al. (2015) found that diversity was higher in liquid fractions, whilst other studies (Veneman et al., 2015; de Mulder et al., 2017) reported higher diversity in solid-phase samples. Another study (Ji et al., 2017) found no difference between fractions when assessing diversity using Simpson Index, but noted that diversity was mainly affected by diet. In the present study *Prevotella* predominated in the liquid phase, reducing the richness and diversity of the overall community. Tapio et al. (2016) reported that buccal swabs could be used to obtain a representative sample of the rumen microbial community when compared with rumen liquid-phase or strained rumen bolus samples. Another study (Ji et al., 2017) found that diet had a greater influence on diversity than rumen fraction. Both these studies used solid samples that had been squeezed to remove the liquid fraction. These strained bolus samples can effectively be classed as a liquid sample as no attempt was made to assess attached solid-associated microbes. de Mulder et al. (2017) reported that crude liquid rumen samples (liquid sample plus small particles of feed that remained after filtering through layers of cheesecloth) provided a good representation of free living bacteria and archaea, but was not a good representation of the rumen ecosystem as a whole.

In conclusion, the rumen microbiota differed between the two contrasting diets, in particular there were marked differences in abundances of Proteobacteria. This is potentially a result of stresses on the microbiome associated with feeding clover. Although many of the same taxonomic group are present, sampling technique (liquid- or solid-phase samples) affects the distribution of abundances. The distribution of OTUs were consistent with the liquid-phase acting as a reservoir for inoculation of solid-phase material. Archaea were more abundant in the solid samples, suggesting that liquid-phase sampling may not give a full picture of the relationship between the rumen microbiota and methane emissions. It is therefore essential that phase of the rumen is standardized between studies to ensure comparable results.

## Author Contributions

JB and MM carried out laboratory work. JB, PC, RD, MM, and SL analyzed the data. JB and RD prepared the manuscript. All authors reviewed the manuscript.

## Conflict of Interest Statement

The authors declare that the research was conducted in the absence of any commercial or financial relationships that could be construed as a potential conflict of interest.

## Footnotes

1 https://sourceforge.net/projects/bbmap/

2 https://github.com/jbowensruc/PerlScriptFrontiers

3 http://greengenes.lbl.gov/

## Supplementary Material

FIGURE S1 Taxa summary plot of samples at the family level. LC, liquid clover; LG, liquid grass; SC, solid clover; SG, solid grass.

TABLE S1 Relative abundance of taxa from phylum to genus.

TABLE S2 Comparison of genera between phases and diets assessed using Kruskal-Wallis test.

TABLE S3 Results from presence vs. absence analysis.
