# Supplementary figures and images for: Evaluation of Microbial Communities Associated With the Liquid and Solid Phases of the Rumen of Cattle Offered a Diet of Perennial Ryegrass or White Clover
Source: Front Microbiol. 2018 Oct 8;9:2389. doi: 10.3389/fmicb.2018.02389 (PMC6186844; doi:10.3389/fmicb.2018.02389)

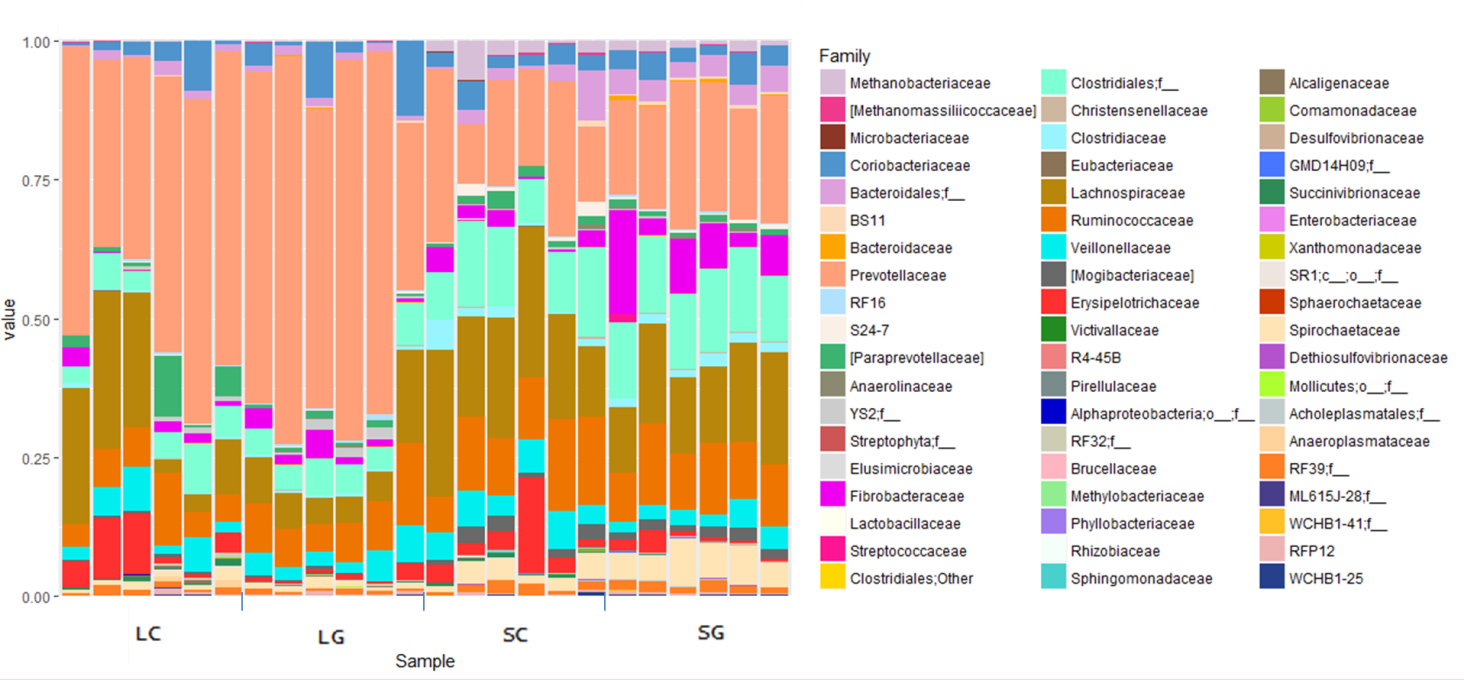

Supplement: FIGURE S1 — Taxa summary plot of samples at the family level. LC, liquid clover; LG, liquid grass; SC, solid clover; SG, solid grass. [file Image_1.TIF]
